# Multimorbidity and risk of atrial fibrillation in the Lifelines cohort

**DOI:** 10.1093/ehjopen/oeaf164

**Published:** 2025-12-13

**Authors:** Colinda van Deutekom, Liann I Weil, Melissa E Middeldorp, Michelle Samuel, Bastiaan Geelhoed, Marieke J H Velt, Victor W Zwartkruis, Denise J C Hanssen, Barbara C Van Munster, Richard C Oude Voshaar, Isabelle C Van Gelder, Michiel Rienstra

**Affiliations:** Department of Cardiology, University of Groningen, University Medical Centre Groningen, P.O. Box 30.001, 9700 RB Groningen, The Netherlands; Department of Internal Medicine, University Centre for Geriatric Medicine, University Medical Centre Groningen, P.O. Box 30.001, 9700 RB Groningen, The Netherlands; Department of Cardiology, University of Groningen, University Medical Centre Groningen, P.O. Box 30.001, 9700 RB Groningen, The Netherlands; Department of Cardiology, University of Groningen, University Medical Centre Groningen, P.O. Box 30.001, 9700 RB Groningen, The Netherlands; Department of Epidemiology, University of Groningen, University Medical Centre Groningen, P.O. Box 30.001, 9700 RB Groningen, The Netherlands; Department of Medicine, Dalhousie University, Halifax, NS, Canada; Department of Cardiology, University of Groningen, University Medical Centre Groningen, P.O. Box 30.001, 9700 RB Groningen, The Netherlands; Department of Cardiology, University of Groningen, University Medical Centre Groningen, P.O. Box 30.001, 9700 RB Groningen, The Netherlands; Department of Cardiology, University of Groningen, University Medical Centre Groningen, P.O. Box 30.001, 9700 RB Groningen, The Netherlands; Department of Psychiatry, University of Groningen, University Medical Centre Groningen, P.O. Box 30.001, 9700 RB Groningen, The Netherlands; Department of Internal Medicine, University Centre for Geriatric Medicine, University Medical Centre Groningen, P.O. Box 30.001, 9700 RB Groningen, The Netherlands; Department of Psychiatry, University of Groningen, University Medical Centre Groningen, P.O. Box 30.001, 9700 RB Groningen, The Netherlands; Department of Cardiology, University of Groningen, University Medical Centre Groningen, P.O. Box 30.001, 9700 RB Groningen, The Netherlands; Department of Cardiology, University of Groningen, University Medical Centre Groningen, P.O. Box 30.001, 9700 RB Groningen, The Netherlands

**Keywords:** Atrial fibrillation, Multimorbidity, Comorbidity, Risk factors, Clustering

## Abstract

**Aims:**

Associations of individual comorbidities with incident atrial fibrillation (AF) are well-studied. However, the impact of multimorbidity and potentially clustering of comorbidities on incident AF remains unclear. This study investigated the number and clustering of (non-)cardiovascular comorbidities with incident AF.

**Methods and results:**

We studied 25 (non-)cardiovascular comorbidities in 76 648 participants from the Lifelines cohort. Logistic regression was used to study the association between the number of comorbidities and incident AF. Latent class analysis was used to identify comorbidity clusters. Mean age was 46.4 ± 2.6 years and 59.3% were women. In this population, 56 034 (73.1%) participants had ≥2 comorbidities, 42 575 (55.5%) ≥ 2 cardiovascular comorbidities, and 14 612 (19.1%) ≥ 2 non-cardiovascular comorbidities. After a mean follow-up of 3.70 ± 0.95 years, 188 (0.2%) participants developed incident AF. After adjusting for age and sex, the total number of comorbidities (OR 1.10 [1.01–1.19], *P* = 0.022) and number of cardiovascular comorbidities (OR 1.18 [1.06–1.31], *P* = 0.002) were associated with incident AF, but not the number of non-cardiovascular comorbidities. We identified 12 comorbidity clusters carrying different risks of incident AF (AF incidence rate range 0.00 to 0.58 per 100 person-years, *P* < 0.001) with the median number of comorbidities ranging from one to seven. However, the clusters did not demonstrate specific combinations of comorbidities.

**Conclusion:**

There was a dose-dependent relationship between the number of total comorbidities and cardiovascular comorbidities and risk of incident AF, but not for non-cardiovascular comorbidities. We identified 12 comorbidity clusters with different risks of incident AF; however, these clusters were determined by the number of comorbidities rather than specific combinations.

## Graphical Abstract

**Graphical Abstract:**
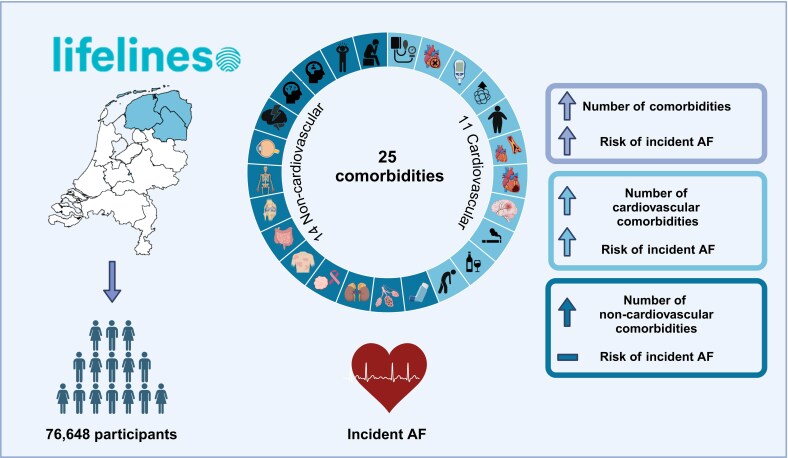

## Introduction

Atrial fibrillation (AF) is a condition that generally occurs in the presence of comorbidities. Multimorbidity, defined as the presence of two or more chronic conditions, is very common among patients with AF especially with increasing age, with ∼80% of patients with AF having at least two comorbidities.^1^ Over the last decades, both the incidence of AF and the comorbidity burden present at the time of AF diagnosis have increased.^2^ Associations of single comorbidities and incident AF are well-studied, and the latest European Society of Cardiology guidelines provide clear recommendations for management of each comorbidity.^3^ However, clear guidance on how to manage multimorbidity is not provided, since evidence is lacking. A small number of observational studies demonstrated an association between cardiovascular multimorbidity and incident AF.^4,5^ Nevertheless, it remains unclear whether a dose-dependent relationship between multimorbidity and incident AF exists, what the role is of non-cardiovascular comorbidities, and whether clustering of certain comorbidities occurs and if it is of importance.

In the present study of the Lifelines cohort, we aimed to investigate (i) the association between the total number of comorbidities, cardiovascular comorbidities, and non-cardiovascular comorbidities with incident AF and (ii) the presence of comorbidity clusters and their association with incident AF.

## Methods

### The Lifelines cohort design and study population

The design and rationale of the Lifelines cohort study have been described in detail elsewhere.^6^ In short, Lifelines is a prospective population-based cohort study, designed to investigate health and health-related behaviour in three generations in the northern part of the Netherlands to obtain insight into healthy ageing. Lifelines participants were recruited through general practitioners and were asked to also invite their family members to participate. Other interested individuals in the northern part of the Netherlands that were not invited via their general practitioner could register directly via the Lifelines website. Together, this resulted in a total of 167 729 participants in the Lifelines general population cohort, with planned long-term follow-up.

Through general assessments every 5 years, Lifelines collects multidisciplinary data via questionnaires, clinical tests, and biological samples from the cohort. The baseline assessment was performed between 2007 and 2013, the second assessment between 2014 and 2017, and the third assessment between 2019 and 2023. The fourth assessment is currently ongoing. The Lifelines cohort study was conducted in accordance with the Declaration of Helsinki and the University Medical Centre Groningen research code.

**Figure 1:**
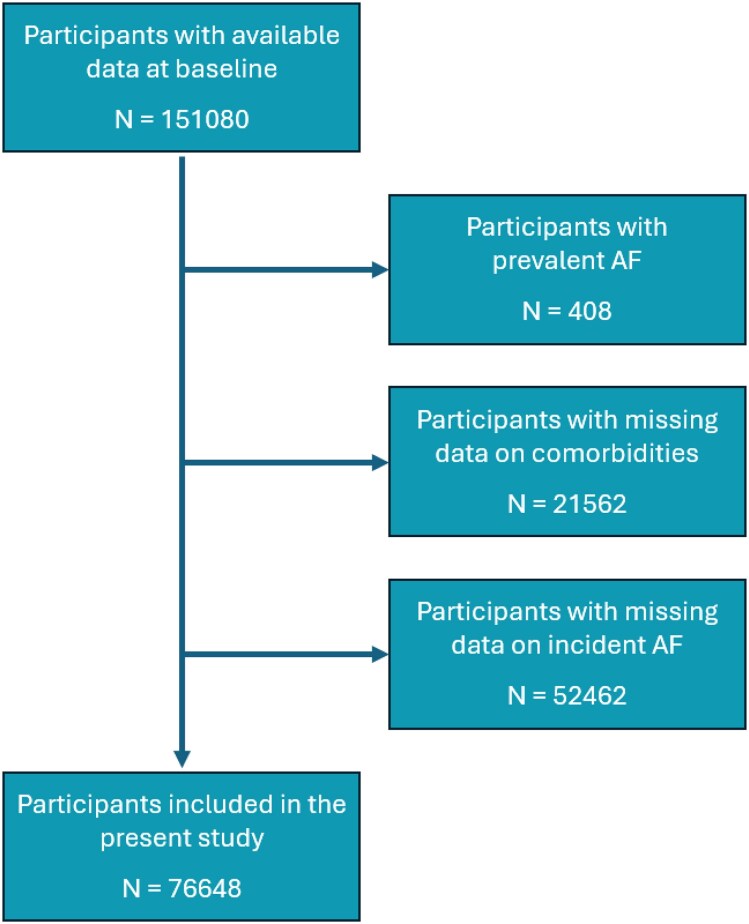
Flowchart of the study population.

For the current study, we used data from the first and second assessment. We included 151 080 participants aged 18 years and over with available data at the first assessment (baseline). Participants without follow-up ECG data were excluded from the analysis, primarily due to non-attendance at the second study visit during the ECG assessment window. This may introduce selection bias; however, baseline characteristics of the included population were largely representative of the overall cohort. We excluded 408 participants because of prevalent AF (self-reported history of AF or the presence of AF on baseline ECG), 21 562 because of missing data on comorbidities, and 52 462 because of missing data on incident AF. The final cohort for the present study included 76 648 participants (*Figure 1*).

### Data collection

All participants were asked to complete a self-administered questionnaire on medical history, use of medication, and lifestyle. Participants were then invited to 1 of the 12 local research sites for clinical measurements, performed by a trained research nurse. A 12-lead resting ECG was made using CardioPerfect software (Welch Allyn DT100) with interpretation by Welch Allyn automated algorithms.^7^ Spirometry was performed following American Thoracic Society guidelines using Welch Allyn V1.6.0.489, PC-based SpiroPerfect with CardioPerfect Workstation Software.

### Definitions of comorbidities and comorbidity groups

At the baseline assessment, the presence of 25 risk factors and comorbidities was assessed (see Supplementary material online, *Appendix I*). Cardiovascular comorbidities included hypertension, heart failure, diabetes mellitus, hypercholesterolaemia, obesity, coronary heart disease, previous myocardial infarction, previous stroke, smoking status, alcohol use, and physical activity. Non-cardiovascular comorbidities included chronic obstructive pulmonary disease (COPD), asthma, kidney disease, cancer, psoriasis, inflammatory bowel disease, rheumatoid arthritis, osteoarthritis, visual impairment, migraine, dementia, schizophrenia, anxiety, and depression.

The population was divided into groups based on the number of comorbidities present at baseline (0–1 comorbidities vs. ≥2 comorbidities). Furthermore, the population was divided based on the number of cardiovascular comorbidities present (0–1 cardiovascular comorbidities vs. ≥2 cardiovascular comorbidities) and based on the number of non-cardiovascular comorbidities present (0–1 non-cardiovascular comorbidities vs. ≥2 non-cardiovascular comorbidities).

### Definition of atrial fibrillation

The study outcome of incident AF was defined as the presence of AF on the 12-lead resting ECG using an automated interpretation algorithm (Welch Allyn DT100) during the first follow-up moment of the Lifelines study (assessment 2).

### Statistical analyses

Participant characteristics were presented as mean ± standard deviation for normally distributed continuous variables, as median (interquartile range) for not normally distributed continuous variables and as number (percentage) for categorical variables. Differences in incident AF between participants with 0–1 comorbidities vs. ≥2 comorbidities, 0–1 cardiovascular comorbidities vs. ≥2 cardiovascular comorbidities, and 0–1 non-cardiovascular comorbidities vs. ≥2 non-cardiovascular comorbidities at Lifelines cohort entry were assessed using the χ^2^ test. AF incidence rates per 100 person-years were calculated for the different groups. Logistic regression was used to study the association between the number of comorbidities, number of cardiovascular comorbidities, number of non-cardiovascular comorbidities and incident AF, as a continuous variable (number of comorbidities), and as a binary variable (≥2 comorbidities vs. 0–1 comorbidities). Additional logistic regression analyses were performed for the association between the individual comorbidities and incident AF, all unadjusted and adjusted for age and sex. Age was considered a potential effect modifier in the relationship between multimorbidity and AF; therefore, we performed an additional subgroup analysis stratified by age tertiles. Latent class analysis was performed using the aforementioned 25 risk factors and comorbidities and the study outcome incident AF, according to the method by Lanza *et al.*^8^ A priori, the optimum number of latent classes was not known. This was determined by the number of classes for which the Bayesian information criterion reached a minimum value. The C-statistic was examined to assess the goodness of fit of the model. The AF incident rates were calculated for each comorbidity cluster. The analyses were performed using R package (version 4.2.0) and IBM SPSS Statistics 28. The significance level was set at *P* < 0.05.

## Results

### Participant characteristics

**Figure 2:**
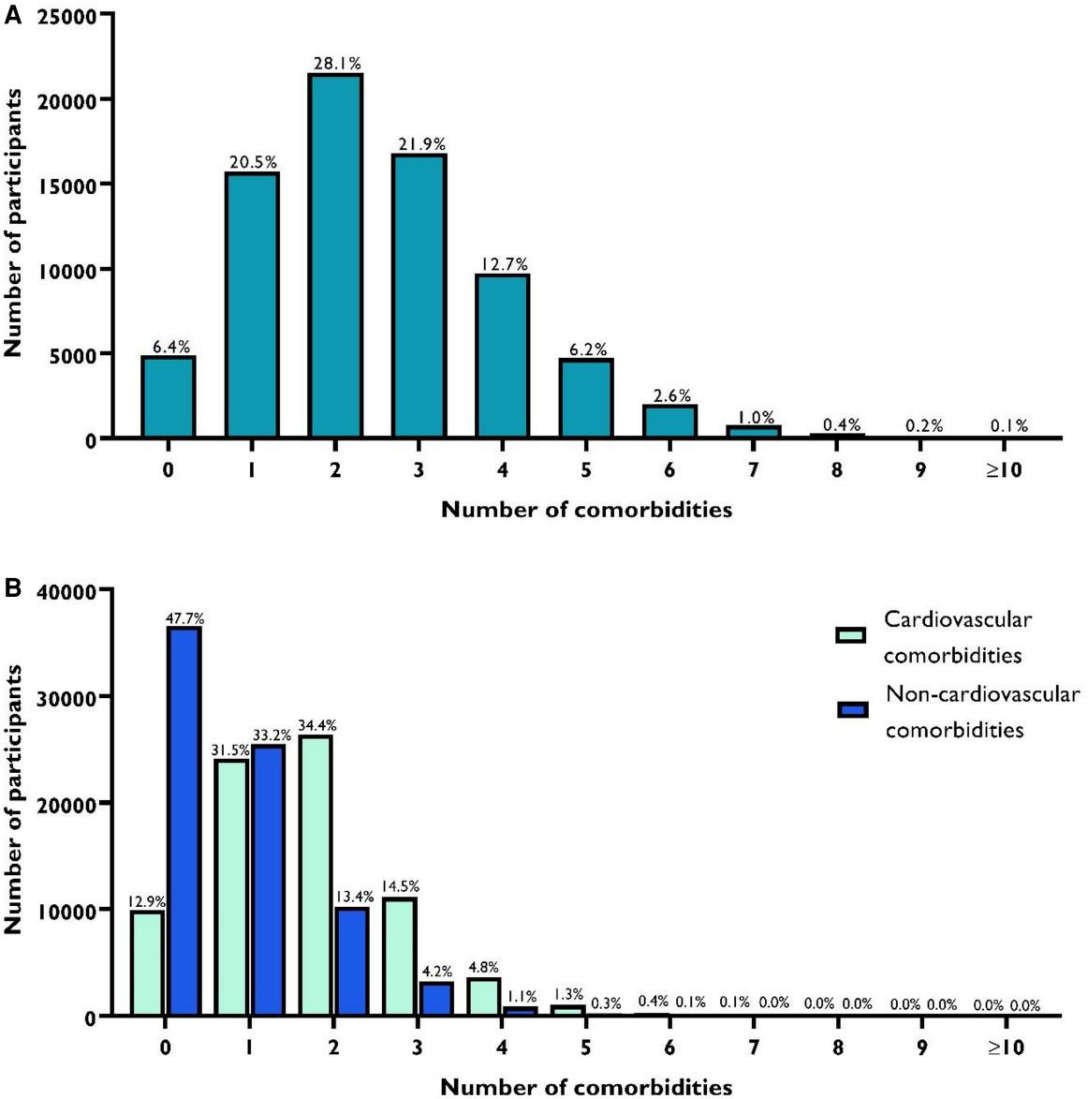
Distribution of comorbidities in the study population. (*A*) Histogram showing the total number of participants by total number of comorbidities (cardiovascular and non-cardiovascular combined). (*B*) Histograms showing the number of participants by number of cardiovascular comorbidities and non-cardiovascular comorbidities, plotted separately. Note that participants may appear in both cardiovascular and non-cardiovascular categories depending on their comorbidity profile. For example, a participant with 1 cardiovascular and 1 non-cardiovascular comorbidity would be represented in both distributions in panel *B* and counted as having 2 comorbidities in panel *A*. Participants with zero comorbidities had neither cardiovascular nor non-cardiovascular conditions.

At baseline, mean age was 46.4 ± 2.6 years and 59.3% were women. The median number of comorbidities in this population was 2 (1–3), with a median of 2 (1–2) cardiovascular comorbidities and 1 (0–1) non-cardiovascular comorbidities (*Table 1*). In this population, 56 034 (73.1%) participants had ≥2 comorbidities, 42 575 (55.5%) ≥ 2 cardiovascular comorbidities, and 14 612 (19.1%) ≥ 2 non-cardiovascular comorbidities. The distribution of the number of comorbidities is shown in *Figure 2*. After a mean follow-up of 3.70 ± 0.95 years, 188 (0.2%) participants developed incident AF.

**Table 1:** Participant characteristics

Characteristic	Total population (*n* = 76 648)
Age (years)	46.4 ± 12.6
Women	45 460 (59.3%)
Number of risk factors and comorbidities	2 (1–3)
Cardiovascular	2 (1–2)
Non-cardiovascular	1 (0–1)
≥2 Comorbidities	56 034 (73.1%)
≥2 Cardiovascular comorbidities	42 575 (55.5%)
≥2 Non-cardiovascular comorbidities	14 612 (19.1%)
Hypertension	13 843 (18.1%)
Heart failure	505 (0.7%)
Diabetes mellitus	1718 (2.2%)
Hypercholesterolaemia	12 457 (16.3%)
Obesity	10 848 (14.2%)
Coronary heart disease	1318 (1.7%)
Previous myocardial infarction	771 (1.0%)
Previous stroke	515 (0.7%)
Smoking status	
Never	36 077 (47.1%)
Ex	28 905 (37.7%)
Current	11 666 (15.2%)
Alcohol use	
≤1 glass/day	26 583 (34.7%)
2 glasses/day	27 950 (36.5%)
≥3 glasses/day	22 115 (28.9%)
Physical activity	
0–1 days/week	9908 (12.9%)
2–4 days/week	28 525 (37.2%)
5–7 days/week	38 215 (49.9%)
COPD	2959 (3.9%)
Asthma	6076 (7.9%)
Kidney disease	1793 (2.3%)
Cancer	3771 (4.9%)
Psoriasis	2229 (2.9%)
Inflammatory bowel disease	688 (0.9%)
Rheumatoid arthritis	1575 (2.1%)
Osteoarthritis	6385 (8.3%)
Visual impairment	13 744 (17.9%)
Migraine	14 003 (18.3%)
Dementia	<10 (−%)
Schizophrenia	55 (0.1%)
Anxiety	5334 (7.0%)
Depression	2006 (2.6%)

Cardiovascular comorbidities include hypertension, heart failure, diabetes mellitus, hypercholesterolaemia, obesity, coronary heart disease, previous myocardial infarction, previous stroke, smoking status, alcohol use, and physical activity. Non-cardiovascular comorbidities include COPD, asthma, kidney disease, cancer, psoriasis, inflammatory bowel disease, rheumatoid arthritis, osteoarthritis, visual impairment, migraine, dementia, schizophrenia, anxiety, and depression. COPD, chronic obstructive pulmonary disease.

### Number of comorbidities and incident AF

**Figure 3:**
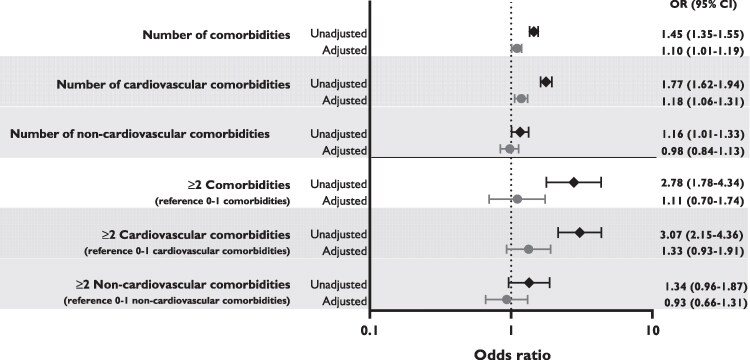
Association between number of comorbidities with incident atrial fibrillation, unadjusted and adjusted for age and sex.

Logistic regression analysis showed that the number of comorbidities, treated as a continuous variable, was statistically significant associated with incident AF (OR 1.45 per additional comorbidity, 95% CI 1.35–1.55, *P* < 0.001), as were the number of cardiovascular comorbidities (OR 1.77 per additional comorbidity, 95% CI 1.62–1.94, *P* < 0.001) and the number of non-cardiovascular comorbidities (OR 1.16 per additional comorbidity, 95% CI 1.01–1.33, *P* = 0.037) (*Figure 3*). After adjusting for age and sex, the association between the number of comorbidities and incident AF remained statistically significant (OR 1.10 per additional comorbidity, 95% CI 1.01–1.19, *P* = 0.022), as well as the association between the number of cardiovascular comorbidities and incident AF (OR 1.18 per additional comorbidity, 95% CI 1.06–1.31, *P* = 0.002). The association between the number of non-cardiovascular comorbidities and incident AF was no longer statistically significant after adjusting for age and sex. The results of the logistic regression analyses with the individual comorbidities and incident AF are shown in Supplementary material online, *Appendix II*.

### Comorbidity groups and incident AF

During follow-up, 166 (0.3%) participants with ≥2 comorbidities developed AF compared to 22 (0.1%) participants with 0–1 comorbidities (*P* < 0.001). Participants with ≥2 cardiovascular comorbidities had a higher incidence of AF compared to participants with 0–1 cardiovascular comorbidities (149 [0.3%] vs. 39 [0.1%], respectively, *P* < 0.001); however, there was no statistically significant difference between participants with ≥2 and 0–1 non-cardiovascular comorbidities regarding incident AF (45 [0.3%] vs. 143 [0.2%], respectively, *P* = 0.091). Logistic regression analysis showed that participants with ≥2 comorbidities had a statistically significant higher risk of incident AF (OR 2.78, 95% CI 1.78–4.34, < 0.001) compared to participants with 0–1 comorbidities, as did participants with ≥2 cardiovascular comorbidities (OR 3.07, 95% CI 2.15–4.36, *P* < 0.001) compared to participants with 0–1 cardiovascular comorbidities. However, these associations did not remain statistically significant after adjusting for age and sex (*Figure 3*). Participants with ≥2 non-cardiovascular comorbidities did not have a statistically significant higher risk of incident AF compared to participants with 0–1 non-cardiovascular comorbidities (OR 1.34, 95% CI 0.96–1.87, *P* = 0.090).

### Age-modifying effect on relation between number of comorbidities and incident AF

In an additional analysis, the population was divided into age tertiles to assess effect modification on the association between number of comorbidities and incident AF (see Supplementary material online, *Appendix III*). Participants in the oldest tertile had more risk factors and comorbidities (3 (2–4)) compared to participants in the younger tertiles (2 (1–3)). There were 167 participants with incident AF in the oldest tertile, compared to 4 participants with incident AF in the youngest tertile and 17 participants with incident AF in the middle tertile. After adjusting for age and sex, the number of cardiovascular comorbidities was statistically significant associated with incident AF in the middle (OR 1.56 (1.07–2.27), *P* = 0.020) and oldest (OR 1.15 (1.03–1.28), *P* = 0.010) tertiles (see Supplementary material online, *Appendix IV*).

### Comorbidity clustering

**Figure 4:**
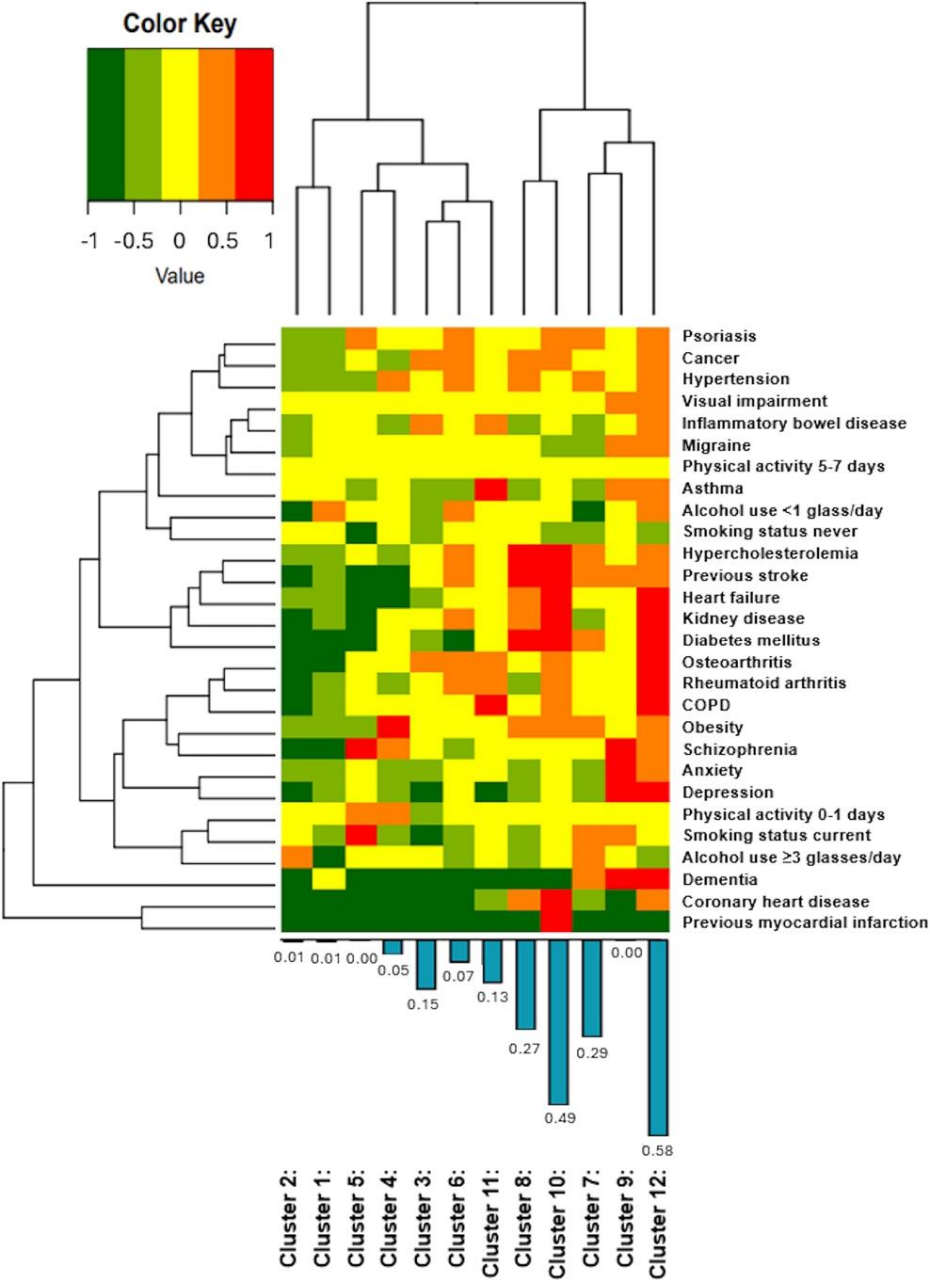
Heat map of the individual risk factors and comorbidities within each comorbidity cluster and atrial fibrillation incidence rates. The colours represent the log relative risk of the presence of the risk factor or comorbidity compared to the average individual in this cohort. The branching diagrams represent the hierarchy of categories based on the degree of similarity between the risk factors/comorbidities (rows) or comorbidity clusters (columns). The bars below the heat map represent the atrial fibrillation incidence rate per 100 person years per cluster.

Participants were clustered based on 25 cardiovascular and non-cardiovascular comorbidities. A model with 12 latent classes provided the best fit, with the lowest Bayesian information criterion value. The C-statistic of this model was 0.764. AF incidence rates varied between the comorbidity clusters, ranging from 0.00 to 0.58 per 100 person years (*P* < 0.001). The cluster with the highest AF incidence rate was cluster 12 with a median of 3 cardiovascular and 3 non-cardiovascular comorbidities. Other clusters with higher AF incidence rates were predominantly determined by presence of cardiovascular comorbidities (*Table 2*, *Figure 4*).

**Table 2:** Characteristics of the population by comorbidity clusters

Characteristic	Cluster 1 *n* = 26 443	Cluster 2 *n* = 15 304	Cluster 3 *n* = 10 543	Cluster 4 *n* = 5125	Cluster 5 *n* = 5013	Cluster 6 *n* = 4133	Cluster 7 *n* = 3237	Cluster 8 *n* = 2164	Cluster 9 *n* = 2052	Cluster 10 *n* = 1209	Cluster 11 *n* = 865	Cluster 12 *n* = 560
Age (years)	44.1 ± 11.3	39.4 ± 11.8	51.8 ± 11.0	45.6 ± 10.9	44.0 ± 10.9	58.6 ± 9.9	51.8 ± 10.3	58.2 ± 10.1	45.0 ± 10.7	62.0 ± 9.7	52.7 ± 8.7	58.9 ± 9.5
Women	18 478 (69.9%)	6068 (39.6%)	6196 (58.8%)	3568 (69.6%)	3164 (63.1%)	3071 (74.3%)	924 (28.5%)	1297 (59.9%)	1535 (74.8%)	262 (21.7%)	482 (55.7%)	415 (74.1%)
Number of risk factors and comorbidities	1 (1–2)	2 (2–3)	3 (2–4)	3 (2–4)	3 (2–4)	3 (3–4)	4 (4–5)	4 (3–5)	4 (4–5)	6 (5–7)	5 (4–6)	7 (6–8)
Cardiovascular	1 (0–1)	2 (1–2)	2 (2–3)	2 (2–3)	2 (2–2)	2 (1–2)	4 (3–4)	3 (3–4)	2 (1–2)	5 (4–6)	2 (1–3)	3 (3–4)
Non-cardiovascular	0 (0–1)	0 (0–1)	1 (0–1)	1 (0–1)	1 (0–2)	2 (1–2)	1 (0–1)	0 (0–1)	3 (2–3)	1 (0–2)	3 (2–3)	3 (3–4)
Hypertension	2624 (9.9%)	1839 (12.0%)	1967 (18.7%)	1450 (28.3%)	241 (4.8%)	1584 (38.3%)	2127 (65.7%)	1005 (46.4%)	270 (13.2%)	346 (28.6%)	222 (25.7%)	168 (30.0%)
Heart failure	75 (0.3%)	33 (0.2%)	30 (0.3%)	<10 (−%)	<10 (−%)	61 (1.5%)	31 (1.0%)	36 (1.7%)	18 (0.9%)	173 (14.3%)	<10 (−%)	43 (7.7%)
Diabetes mellitus	119 (0.5%)	24 (0.2%)	86 (0.8%)	76 (1.5%)	<10 (−%)	<10 (−%)	143 (4.4%)	794 (36.7%)	50 (2.4%)	162 (13.4%)	13 (1.5%)	244 (43.6%)
Hypercholesterolaemia	1447 (5.5%)	526 (3.4%)	2139 (20.3%)	101 (2.0%)	817 (16.3%)	1624 (39.3%)	1737 (53.7%)	2053 (94.9%)	324 (15.8%)	1157 (95.7%)	183 (21.2%)	349 (62.3%)
Obesity	33 (0.1%)	496 (3.2%)	948 (9.0%)	5125 (100.0%)	288 (5.7%)	212 (5.1%)	1448 (44.7%)	890 (41.1%)	550 (26.8%)	275 (22.7%)	194 (22.4%)	389 (69.5%)
Coronary heart disease	<10 (−%)	17 (0.1%)	<10 (−%)	<10 (−%)	<10 (−%)	<10 (−%)	18 (0.6%)	26 (1.2%)	<10 (−%)	1209 (100.0%)	<10 (−%)	29 (5.2%)
Previous myocardial infarction	<10 (−%)	<10 (−%)	<10 (−%)	<10 (−%)	<10 (−%)	<10 (−%)	<10 (−%)	<10 (−%)	<10 (−%)	771 (63.8%)	<10 (−%)	<10 (−%)
Previous stroke	46 (0.2%)	<10 (−%)	34 (0.3%)	<10 (−%)	<10 (−%)	76 (1.8%)	65 (2.0%)	178 (8.2%)	36 (1.8%)	42 (3.5%)	<10 (−%)	15 (2.7%)
Smoking status												
Never	20 668 (78.2%)	7038 (46.0%)	13 (0.1%)	3292 (64.2%)	<10 (−%)	2331 (56.4%)	291 (9.0%)	919 (42.5%)	755 (36.8%)	256 (21.2%)	366 (42.3%)	148 (26.4%)
Ex	5244 (19.8%)	4157 (27.2%)	10 530 (99.9%)	1528 (29.8%)	406 (8.1%)	1561 (37.8%)	2054 (63.5%)	1104 (51.0%)	777 (37.9%)	817 (67.6%)	373 (43.1%)	354 (63.2%)
Current	531 (2.0%)	4109 (26.8%)	<10 (−%)	305 (6.0%)	4607 (91.9%)	241 (5.8%)	892 (27.6%)	141 (6.5%)	520 (25.3%)	136 (11.2%)	126 (14.6%)	58 (10.4%)
Alcohol use												
≤ 1 glass/day	16 068 (60.8%)	<10 (−%)	36 (0.3%)	2895 (56.5%)	1308 (26.1%)	2901 (70.2%)	<10 (−%)	1300 (60.1%)	1000 (48.7%)	428 (35.4%)	311 (36.0%)	336 (60.0%)
2 glasses/day	10 375 (39.2%)	1507 (9.8%)	9317 (88.4%)	1265 (24.7%)	1964 (39.2%)	1080 (26.1%)	296 (9.1%)	724 (33.5%)	507 (24.7%)	459 (38.0%)	310 (35.8%)	146 (26.1%)
≥3 glasses/day	<10 (−%)	13 797 (90.2%)	1190 (11.3%)	965 (18.8%)	1741 (34.7%)	152 (3.7%)	2941 (90.9%)	140 (6.5%)	545 (26.6%)	322 (26.6%)	244 (28.2%)	78 (13.9%)
Physical activity												
0–1 days	3503 (13.2%)	1178 (7.7%)	261 (2.5%)	1178 (23.0%)	2040 (40.7%)	291 (7.0%)	485 (15.0%)	256 (11.8%)	395 (19.2%)	111 (9.2%)	98 (11.3%)	112 (20.0%)
2–4 days	9962 (37.7%)	6550 (42.8%)	3719 (35.3%)	2119 (41.3%)	1756 (35.0%)	850 (20.6%)	1329 (41.1%)	719 (33.2%)	673 (32.8%)	349 (28.9%)	301 (34.8%)	198 (35.4%)
5–7 days	12 978 (49.1%)	7576 (49.5%)	6563 (62.2%)	1828 (35.7%)	1217 (24.3%)	2992 (72.4%)	1423 (44.0%)	1189 (54.9%)	984 (48.0%)	749 (62.0%)	466 (53.9%)	250 (44.6%)
COPD	258 (1.0%)	25 (0.2%)	382 (3.6%)	130 (2.5%)	266 (5.3%)	280 (6.8%)	238 (7.4%)	33 (1.5%)	109 (5.3%)	106 (8.8%)	865 (100.0%)	267 (47.7%)
Asthma	1841 (7.0%)	1464 (9.6%)	262 (2.5%)	647 (12.6%)	109 (2.2%)	160 (3.9%)	95 (2.9%)	59 (2.7%)	316 (15.4%)	64 (5.3%)	864 (99.9%)	195 (34.8%)
Kidney disease	327 (1.2%)	56 (0.4%)	359 (3.4%)	100 (2.0%)	15 (0.3%)	426 (10.3%)	13 (0.4%)	249 (11.5%)	30 (1.5%)	119 (9.8%)	17 (2.0%)	82 (14.6%)
Cancer	744 (2.8%)	145 (0.9%)	909 (8.6%)	121 (2.4%)	246 (4.9%)	941 (22.8%)	167 (5.2%)	183 (8.5%)	97 (4.7%)	110 (9.1%)	43 (5.0%)	65 (11.6%)
Psoriasis	450 (1.7%)	215 (1.4%)	322 (3.1%)	131 (2.6%)	399 (8.0%)	252 (6.1%)	202 (6.2%)	31 (1.4%)	57 (2.8%)	59 (4.9%)	27 (3.1%)	84 (15.0%)
Inflammatory bowel disease	225 (0.9%)	48 (0.3%)	161 (1.5%)	26 (0.5%)	76 (1.5%)	54 (1.3%)	11 (0.3%)	12 (0.6%)	30 (1.5%)	14 (1.2%)	12 (1.4%)	19 (3.4%)
Rheumatoid arthritis	161 (0.6%)	55 (0.4%)	229 (2.2%)	55 (1.1%)	143 (2.9%)	500 (12.1%)	118 (3.6%)	<10 (−%)	63 (3.1%)	45 (3.7%)	30 (3.5%)	172 (30.7%)
Osteoarthritis	156 (0.6%)	90 (0.6%)	1384 (13.1%)	404 (7.9%)	324 (6.5%)	2465 (59.6%)	486 (15.0%)	179 (8.3%)	240 (11.7%)	175 (14.5%)	119 (13.8%)	363 (64.8%)
Visual impairment	3681 (13.9%)	1737 (11.3%)	2253 (21.4%)	655 (12.8%)	1461 (29.1%)	1194 (28.9%)	861 (26.6%)	262 (12.1%)	913 (44.5%)	267 (22.1%)	221 (25.5%)	239 (42.7%)
Migraine	5389 (20.4%)	1448 (9.5%)	1699 (16.1%)	1253 (24.4%)	1245 (24.8%)	1284 (31.1%)	105 (3.2%)	238 (11.0%)	814 (39.7%)	130 (10.8%)	189 (21.8%)	209 (37.3%)
Dementia	<10 (−%)	<10 (−%)	<10 (−%)	<10 (−%)	<10 (−%)	<10 (−%)	<10 (−%)	<10 (−%)	<10 (−%)	<10 (−%)	<10 (−%)	<10 (−%)
Schizophrenia	<10 (−%)	<10 (−%)	<10 (−%)	<10 (−%)	21 (0.4%)	<10 (−%)	<10 (−%)	<10 (−%)	13 (0.6%)	<10 (−%)	<10 (−%)	<10 (−%)
Anxiety	1189 (4.5%)	334 (2.2%)	469 (4.4%)	163 (3.2%)	660 (13.2%)	234 (5.7%)	137 (4.2%)	80 (3.7%)	1787 (87.1%)	92 (7.6%)	47 (5.4%)	142 (25.4%)
Depression	184 (0.7%)	13 (0.1%)	<10 (−%)	65 (1.3%)	150 (3.0%)	80 (1.9%)	58 (1.8%)	<10 (−%)	1337 (65.2%)	33 (2.7%)	<10 (−%)	76 (13.6%)

Cardiovascular comorbidities include hypertension, heart failure, diabetes mellitus, hypercholesterolaemia, obesity, coronary heart disease, previous myocardial infarction, previous stroke, smoking status, alcohol use, and physical activity. Non-cardiovascular comorbidities include COPD, asthma, kidney disease, cancer, psoriasis, inflammatory bowel disease, rheumatoid arthritis, osteoarthritis, visual impairment, migraine, dementia, schizophrenia, anxiety, and depression. COPD, chronic obstructive pulmonary disease.

Cluster 1 was the largest (*n* = 26 443) and consisted predominantly of women with few risk factors and comorbidities. Cluster 2 (*n* = 15 304) was the youngest (mean age 39.4 ± 11.8) and consisted predominantly of men with few risk factors and comorbidities, but a large proportion of alcohol users ≥3 glasses/day. Cluster 3 (*n* = 10 543) contained participants with a moderately higher probability of having osteoarthritis, inflammatory bowel disease, and/or cancer compared to the average participant in the study population. Cluster 4 (*n* = 5125) included obese participants. Cluster 5 (*n* = 5013) contained participants who were more likely to be current smokers and/or have schizophrenia. Cluster 6 (*n* = 4133) consisted of participants with moderately increased risk of having cardiovascular and/or non-cardiovascular risk factors and comorbidities, with a relatively high prevalence of cancer compared to the other clusters. Cluster 7 (*n* = 3237) and Cluster 8 (*n* = 2164) both consisted of participants with mainly cardiovascular risk factors and comorbidities. Cluster 7 included a larger proportion of men, alcohol users, current smokers, and participants with hypertension compared to Cluster 8, while Cluster 8 included predominantly women with more often diabetes mellitus and/or hypercholesterolaemia. Cluster 9 (*n* = 2052) is characterized by a large proportion of participants with comorbidities affecting mental health. Cluster 10 (*n* = 1209) included participants with coronary heart disease, mainly men, with the majority also reporting a previous myocardial infarction and relatively high prevalence of other cardiovascular comorbidities. Cluster 11 (*n* = 865) included participants with pulmonary comorbidities. Cluster 12 was the smallest cluster (*n* = 560) and consisted predominantly of women with relatively high prevalence of risk factors and comorbidities in general (median 7 [6–8]) (*Table 2*, *Figure 4*).

## Discussion

In the Lifelines cohort, we found a dose-dependent relationship between the number of total comorbidities and cardiovascular comorbidities and risk of incident AF, also after adjusting for age and sex. A similar relation was not present for non-cardiovascular comorbidities and incident AF. We identified 12 comorbidity clusters with different risks of incident AF; however, clusters were determined by number of comorbidities rather than specific combinations of comorbidities.

### Number of comorbidities and incident AF

The number of comorbidities was associated with incident AF in the present study, which may suggest a cumulative effect of the comorbidities present on the risk of incident AF. Vanbeselaere *et al.* found that the Charlson Comorbidity Index was positively associated with incident AF,^4^ similar to the association observed between the number of comorbidities and incident AF in the present study. Although there are differences between the Charlson Comorbidity Index and the present study regarding the included comorbidities, and the Charlson Comorbidity Index is based on a weight of the included diseases rather than the number of comorbidities, it can be used as a measure for comorbidity burden. Other studies did not specifically investigate the association between the number of comorbidities and incident AF but described a higher prevalence of comorbidities in patients with incident AF compared to controls, indicative of an association.^9–11^

Regarding the number of cardiovascular comorbidities and incident AF, previous data from the PREVEND cohort including 10 cardiovascular comorbidities demonstrated that more comorbidities were present in groups with higher risk of incident AF.^5^ Similar findings were reported in the Rotterdam Study, which demonstrated the association between the number of cardiometabolic comorbidities and risk of AF.^12^ Additionally results from the present study indicated that this association has a dose-dependent relationship. The positive association between the number of (cardiovascular) comorbidities and incident AF indicates the cumulative effects of having multiple comorbidities on the risk of incident AF, especially cardiovascular comorbidities. Our findings underscore the growing public health challenge posed by the increasing burden of AF in the context of rising multimorbidity. Recent data from the Global Burden of Disease study show that the prevalence of AF and its attributable risk factors including hypertension, obesity, and diabetes has substantially increased.^13^ The dose-dependent relationship we observed between the number of comorbidities and incident AF supports the need for a more integrated, upstream approach to risk factor management. These results suggest that preventive strategies targeting multiple coexisting conditions particularly cardiovascular risk factors may be essential to mitigating the future burden of AF.

### Comorbidity groups vs. number of comorbidities

After adjustment for age and sex, we observed a dose-dependent relationship between the number of comorbidities and incident AF, but there was no increased risk for ≥2 comorbidities vs. 0–1 comorbidities. It is important to consider that the difference in results may be explained by loss of data resulting from dichotomization of the continuous variable. However, other potential explanations should also be taken into account. The observed dose-dependent relationship between number of comorbidities suggests that defining multimorbidity as 2 or more comorbidities simplifies the complex relation between high number of comorbidities and incident AF. It is important to acknowledge that there are substantial differences in the severity of individual comorbidities, with some causing continuous symptoms and impact, while others are intermittently. Additionally, complexity of treatments is also highly variable. Whether individuals with multimorbidity require a completely different management strategy is not only dependent on the presence of multimorbidity, but as we demonstrate, the cumulative number of comorbidities and severity and complexity of treatment also need to be considered.^14^

### Cardiovascular and non-cardiovascular comorbidities in relation to incident AF

Although AF is commonly associated with individual cardiovascular comorbidities, there is increasing evidence of AF also being associated with non-cardiovascular comorbidities.^15–23^ In the present study, we observed a dose-dependent relationship between number of cardiovascular comorbidities and incident AF after adjusting for age and sex. We did not observe a similar association between the number of non-cardiovascular comorbidities and incident AF. There can be various explanations. First, differences in the severity of non-cardiovascular comorbidities, and the differences in complexity of management may play a role. Second, for some non-cardiovascular comorbidities, a clear pathophysiological mechanism is uncertain, and some may be more risk-modifiers than risk factors of incident AF. While chronic kidney disease is a known risk factor for cardiovascular disease and AF,^24^ it was analysed separately from cardiovascular comorbidities in this study to avoid overlapping constructs and to reflect its systemic, multi-organ nature. Future studies may consider combining renal and cardiovascular comorbidity burdens to further explore their joint impact on AF risk.

### Age-modifying effect on relation between comorbidity groups, number of comorbidities, and incident AF

The observed higher risk of incident AF for participants with ≥2 comorbidities compared to 0–1 comorbidities as well as ≥2 cardiovascular comorbidities compared to 0–1 cardiovascular comorbidities was highly statistically significant in the unadjusted model but was attenuated to statistically insignificant levels after adjusting for age and sex. In the subgroup analysis, participants with ≥2 comorbidities compared to 0–1 comorbidities had a higher risk of incident AF only in the oldest age tertile. However, this association was no longer statistically significant after adjustment for age and sex. Only the number of cardiovascular comorbidities remained statistically significantly associated with incident AF in the middle and oldest tertile after adjustment for age and sex. Although the subgroup analyses were limited by the low event rate in the youngest and middle tertiles, it seems that age is an important confounder in the association between multimorbidity and incident AF. Age is known to be a risk factor for multimorbidity as well as incident AF.^25,26^

### Clustering of comorbidities

In our study all comorbidities were weighted equally in the composite comorbidity counts; this approach, while consistent with multimorbidity frameworks, does not account for the differential impact of individual conditions on AF risk. For example, heart failure and hypertension are more strongly associated with AF than conditions such as visual impairment or certain cancers, and lifestyle factors such as alcohol use may have a non-linear or more potent effect than others like smoking. This approach may underrepresent the differential impact of specific conditions on AF risk. As a result, clusters may be more reflective of comorbidity burden than clinically meaningful combinations. However, comorbidities with shared risk factors and/or underlying pathophysiological mechanisms may occur together, i.e. clustering of comorbidities. In this study we used latent class analysis to identify such clusters. We conducted a latent class cluster analysis to identify patterns of co-occurring comorbidities. The 12 resulting clusters reflect distinct multimorbidity profiles. Given the low AF incidence, this analysis was descriptive rather than predictive or causal. Latent class analysis has several advantages. It is performed with dichotomized data, and there are more formal criteria to determine the optimum number of clusters. Latent class analysis has been used before in different cohorts to identify clusters of individuals with similar risk factors and comorbidities.^27–29^ Although most clusters in our study showed a higher prevalence of some comorbidities compared to others, the differences between clusters seems predominantly determined by the number of comorbidities rather than specific combinations of certain comorbidities in different clusters. This is not a unique finding; Whitson *et al.* aimed to define multimorbidity classes based on patterns of comorbidity clustering including 13 conditions.^29^ The authors reported that comorbidity count, rather than patterns of comorbidities, defined their latent classes. We demonstrated that identified clusters had different risks of incident AF, where the risk of incident AF was the highest among clusters determined by a high number of (cardiovascular) comorbidities. This is in line with previous literature as cardiovascular comorbidities are known to increase the risk of incident AF.^9,30^

Few other studies investigated the association between clusters and the risk of incident AF using latent class analysis. While national databases may offer larger samples and continuous follow-up, the Lifelines cohort provides detailed, prospective data on comorbidities and lifestyle factors. This enables a nuanced exploration of multimorbidity patterns in relation to AF risk, which may be less feasible in registry-based studies. Previous clustering approaches in the PREVEND population-based cohort focusing on cardiovascular comorbidities did not find clusters with clear combinations of comorbidities but found clusters with different number of comorbidities carrying different risks of incident AF, comparable to the present study.^5,31^

### Limitations

The main strengths of the present study are the use of data from a large community-based population and inclusion of a large and diverse selection of both cardiovascular and non-cardiovascular comorbidities. However, this study also has some limitations that need to be considered. The presence of risk factors and comorbidities was mainly based on self-reported data, which may have caused over- or underreporting. Furthermore, our list of comorbidities and risk factors, while extensive, is not exhaustive (see Supplementary material online, *Appendix V*). Recent literature has highlighted the relevance of non-traditional or emerging risk factors for AF, such as environmental exposures, autonomic dysfunction, and psychosocial stressors, which may act as potential confounders or modulators of AF risk but were not captured in our dataset.^32^ Information on the severity or grading of individual comorbidities was not available. Medication data were limited, lacking details on indication, dosage, adherence, and duration, and no information was available on comorbidity severity or control. Therefore, we could not assess the modifying effects of treatment or disease control on AF risk. A key limitation of our study is the reliance on a single 12-lead ECG at the scheduled follow-up visit to detect incident AF. This approach likely underestimates the true incidence of AF, particularly among individuals with paroxysmal or asymptomatic AF who may have been in sinus rhythm during the visit. Self-reported or ICD-coded AF data, as well as continuous or intermittent monitoring (e.g. via wearables or ambulatory ECG), were not available in this cohort. As a result, many transient or subclinical AF episodes may have been missed, which likely contributed to the low observed event rate and may limit the generalizability of the findings. This under detection could also attenuate the observed associations,^33^ especially for comorbidities associated with paroxysmal AF. We were therefore unable to distinguish between symptomatic and asymptomatic cases or determine the timing of AF onset. To reduce bias from missing data, we excluded participants with incomplete information on AF status or comorbidities at baseline or follow-up. As information on the excluded participants was not available for analysis, we were unable to conduct sensitivity analyses comparing them to the included population. The small number of incident AF events limited the analyses stratified by age. The low incidence of new-onset AF likely reflects the cohort’s younger baseline age and short follow-up, potentially amplifying the impact of high-risk comorbidities such as heart failure and CAD. Moreover, the relatively large number of risk factors and comorbidities included in the latent class analysis may potentially have resulted in a reduced clustering efficiency. Mortality data were not available for this analysis, as linkage to national death registries was not accessible at the time of the study. Therefore, we were unable to account for deaths as competing risks or assess loss to follow-up due to mortality. This may have led to underestimation of AF risk in older or higher-risk participants and limits the ability to fully account for competing events in the modelling of incident AF. Lastly, the Lifelines cohort population is predominantly white which limits the generalizability of the results, and the results were not externally validated.

## Conclusion

There was a dose-dependent relationship between the number of total comorbidities and cardiovascular comorbidities and risk of incident AF, but not for non-cardiovascular comorbidities. We identified 12 comorbidity clusters with different risks of incident AF; however, these clusters were determined by the number of comorbidities rather than specific combinations of comorbidities. Awareness of multimorbidity and especially high comorbidity burden is therefore important. Whether multimorbidity management requires a specific approach to reduce the risk of AF warrants further research.

## Lead author biography


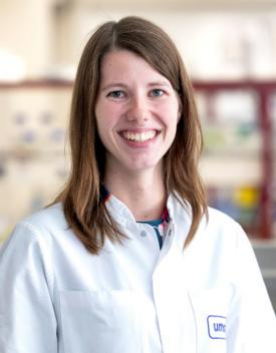


Colinda van Deutekom is a research fellow at the Department of Cardiology, University Medical Center Groningen, The Netherlands. She is working on her doctoral thesis regarding multimorbidity in atrial fibrillation and study coordinator of the EHRA-PATHS cluster randomized trial as part of the Addressing multimorbidity in elderly atrial fibrillation patients through interdisciplinary, patient-centred, systematic care pathways (EHRA-PATHS) project.

## Supplementary Material

oeaf164_Supplementary_Data

## Data Availability

Data may be obtained from a third party and are not publicly available. Researchers can apply to use the Lifelines data used in this study.

## References

[1] Jani BD, Nicholl BI, McQueenie R, Connelly DT, Hanlon P, Gallacher KI, Lee D, Mair FS. Multimorbidity and co-morbidity in atrial fibrillation and effects on survival: findings from UK Biobank cohort. Europace 2018;20:f329–ff36. 29112751 10.1093/europace/eux322 PMC6277149

[2] Wu J, Nadarajah R, Nakao YM, Nakao K, Wilkinson C, Mamas MA, Camm AJ, Gale CP. Temporal trends and patterns in atrial fibrillation incidence: a population-based study of 3·4 million individuals. Lancet Reg Health Eur 2022;17:100386. 35721699 10.1016/j.lanepe.2022.100386 PMC9198843

[3] Van Gelder IC, Rienstra M, Bunting KV, Casado-Arroyo R, Caso V, Crijns HJGM, De Potter TJR, Dwight J, Guasti L, Hanke T, ESC Scientific Document Group. 2024 ESC Guidelines for the management of atrial fibrillation developed in collaboration with the European Association for Cardio-Thoracic Surgery (EACTS): developed by the task force for the management of atrial fibrillation of the European Society of Cardiology (ESC), with the special contribution of the European Heart Rhythm Association (EHRA) of the ESC. Endorsed by the European Stroke Organisation (ESO). Eur Heart J 2024;45:3314–3414. 39210723 10.1093/eurheartj/ehae176

[4] Vanbeselaere V, Truyers C, Elli S, Buntinx F, De Witte H, Degryse J, Henrard S, Vaes B. Association between atrial fibrillation, anticoagulation, risk of cerebrovascular events and multimorbidity in general practice: a registry-based study. BMC Cardiovasc Disord 2016;16:61. 27021333 10.1186/s12872-016-0235-1 PMC4810573

[5] Van Deutekom C, Geelhoed B, Van Munster BC, Bakker SJL, Gansevoort RT, Van Gelder IC, Rienstra M. Cardiovascular and renal multimorbidity increase risk of atrial fibrillation in the PREVEND cohort. Open Heart 2023;10:e002315. 37460268 10.1136/openhrt-2023-002315 PMC10357795

[6] Scholtens S, Smidt N, Swertz MA, Bakker SJ, Dotinga A, Vonk JM, van Dijk F, van Zon SKR, Wijmenga C, Wolffenbuttel BHR, Stolk RP. Cohort Profile: LifeLines, a three-generation cohort study and biobank. Int J Epidemiol 2014;44:1172–1180. 25502107 10.1093/ije/dyu229

[7] Allyn W. MEANS ECG Physicians’ Manual for Welch Allyn CP Series Electrocardiographs. 2016.

[8] Lanza ST, Tan X, Bray BC. Latent class analysis with distal outcomes: a flexible model-based approach. Struct Equ Modeling 2013;20:1–26. 25419096 10.1080/10705511.2013.742377 PMC4240499

[9] Vermond RA, Geelhoed B, Verweij N, Tieleman RG, Van der Harst P, Hillege HL, Van Gilst WH, Van Gelder IC, Rienstra M. Incidence of atrial fibrillation and relationship with cardiovascular events, heart failure, and mortality: a community-based study from The Netherlands. J Am Coll Cardiol 2015;66:1000–1007. 26314526 10.1016/j.jacc.2015.06.1314

[10] Chao TF, Chiang CE, Chen TJ, Liao JN, Tuan TC, Chen SA. Clinical risk score for the prediction of incident atrial fibrillation: derivation in 7 220 654 Taiwan patients with 438 930 incident atrial fibrillations during a 16-year follow-up. J Am Heart Assoc 2021;10:e020194. 34459227 10.1161/JAHA.120.020194 PMC8649261

[11] Jung M, Yang P-S, Kim D, Sung J-H, Jang E, Yu HT, Kim T-H, Uhm J-S, Pak H-N, Lee M-H, Joung B. Multimorbidity in atrial fibrillation for clinical implications using the Charlson Comorbidity Index. Int J Cardiol 2024;398:131605. 38000669 10.1016/j.ijcard.2023.131605

[12] Lu Z, Ntlapo N, Tilly MJ, Geurts S, Aribas E, Ikram MK, de Groot NMS, Kavousi M. Burden of cardiometabolic disorders and lifetime risk of new-onset atrial fibrillation among men and women: the Rotterdam Study. Eur J Prev Cardiol 2024;31:1141–1149. 38307013 10.1093/eurjpc/zwae045

[13] Dong XJ, Wang BB, Hou FF, Jiao Y, Li HW, Lv SP, Li F-H. Global burden of atrial fibrillation/atrial flutter and its attributable risk factors from 1990 to 2019. Europace 2023;25:793–803. 36603845 10.1093/europace/euac237 PMC10062373

[14] van Deutekom C, Hendriks JML, Myrstad M, Van Gelder IC, Rienstra M. Managing elderly patients wtih atrial fibrillation and multimorbidity: call for a systematic approach. Expert Rev Cardiovasc Ther 2024;22:523–536. 39441182 10.1080/14779072.2024.2416666

[15] Guha A, Fradley MG, Dent SF, Weintraub NL, Lustberg MB, Alonso A, Addison D. Incidence, risk factors, and mortality of atrial fibrillation in breast cancer: a SEER-Medicare analysis. Eur Heart J 2021;43:300–312. 10.1093/eurheartj/ehab745 PMC8914878 34791123

[16] Jakobsen CB, Lamberts M, Carlson N, Lock-Hansen M, Torp-Pedersen C, Gislason GH, Schou M. Incidence of atrial fibrillation in different major cancer subtypes: a Nationwide population-based 12 year follow up study. BMC Cancer 2019;19:1105. 31726997 10.1186/s12885-019-6314-9 PMC6854796

[17] Vinter N, Christesen AMS, Fenger-Grøn M, Tjønneland A, Frost L. Atrial fibrillation and risk of cancer: a Danish population-based cohort study. J Am Heart Assoc 2018;7:e009543. 30371150 10.1161/JAHA.118.009543 PMC6201425

[18] Konecny T, Park JY, Somers KR, Konecny D, Orban M, Soucek F, Parker KO, Scanlon PD, Asirvatham SJ, Brady PA, Rihal CS. Relation of chronic obstructive pulmonary disease to atrial and ventricular arrhythmias. Am J Cardiol 2014;114:272–277. 24878126 10.1016/j.amjcard.2014.04.030

[19] Grymonprez M, Vakaet V, Kavousi M, Stricker BH, Ikram MA, Heeringa J, Franco OH, Brusselle GG, Lahousse L. Chronic obstructive pulmonary disease and the development of atrial fibrillation. Int J Cardiol 2019;276:118–124. 30268382 10.1016/j.ijcard.2018.09.056

[20] Rehm M, Rothenbacher D, Iacoviello L, Costanzo S, Tunstall-Pedoe H, Fitton CA, Söderberg S, Hultdin J, Salomaa V, Jousilahti P, Palosaari T, Kuulasmaa K, Waldeyer C, Schnabel RB, Zeller T, Blankenberg S, Koenig W. Chronic kidney disease and risk of atrial fibrillation and heart failure in general population-based cohorts: the BiomarCaRE project. ESC Heart Fail 2022;9:57–65. 34825788 10.1002/ehf2.13699 PMC8788046

[21] Kim SM, Jeong Y, Kim YL, Kang M, Kang E, Ryu H, Kim Y, Han SS, Ahn C, Oh K-H. Association of chronic kidney disease with atrial fibrillation in the general adult population: a nationwide population-based study. J Am Heart Assoc 2023;12:e028496. 37066806 10.1161/JAHA.122.028496 PMC10227274

[22] Tilly MJ, Geurts S, Zhu F, Bos MM, Ikram MA, de Maat MPM, de Groot NMS, Kavousi M. Autoimmune diseases and new-onset atrial fibrillation: a UK Biobank study. EP Europace 2022;25:804–811. 10.1093/europace/euac244 PMC10062304 36546587

[23] Ahlehoff O, Gislason GH, Jørgensen CH, Lindhardsen J, Charlot M, Olesen JB, Abildstrøm SZ, Skov L, Torp-Pedersen C, Hansen PR. Psoriasis and risk of atrial fibrillation and ischaemic stroke: a Danish Nationwide Cohort Study. Eur Heart J 2011;33:2054–2064. 21840930 10.1093/eurheartj/ehr285

[24] Ha JT, Freedman SB, Kelly DM, Neuen BL, Perkovic V, Jun M, Badve SV. Kidney function, albuminuria, and risk of incident atrial fibrillation: a systematic review and meta-analysis. Am J Kidney Dis 2024;83:350–359.e1. 37777059 10.1053/j.ajkd.2023.07.023

[25] Benjamin EJ, Levy D, Vaziri SM, D'Agostino RB, Belanger AJ, Wolf PA. Independent risk factors for atrial fibrillation in a population-based cohort: the Framingham Heart Study. JAMA 1994;271:840–844. 8114238

[26] Bisquera A, Turner EB, Ledwaba-Chapman L, Dunbar-Rees R, Hafezparast N, Gulliford M, Durbaba S, Soley-Bori M, Fox-Rushby J, Dodhia H, Ashworth M, Wang Y. Inequalities in developing multimorbidity over time: a population-based cohort study from an urban, multi-ethnic borough in the United Kingdom. Lancet Reg Health Eur 2022;12:100247. 34901910 10.1016/j.lanepe.2021.100247 PMC8640725

[27] Olaya B, Moneta MV, Caballero FF, Tyrovolas S, Bayes I, Ayuso-Mateos JL, Haro JM. Latent class analysis of multimorbidity patterns and associated outcomes in Spanish older adults: a prospective cohort study. BMC Geriatr 2017;17:186. 28821233 10.1186/s12877-017-0586-1 PMC5563011

[28] Roomaney RA, van Wyk B, Cois A, Pillay van-Wyk V. Multimorbidity patterns in South Africa: a latent class analysis. Front Public Health 2023;10:1082587. 36711391 10.3389/fpubh.2022.1082587 PMC9875075

[29] Whitson HE, Johnson KS, Sloane R, Cigolle CT, Pieper CF, Landerman L, Hastings SN. Identifying patterns of multimorbidity in older Americans: application of latent class analysis. J Am Geriatr Soc 2016;64:1668–1673. 27309908 10.1111/jgs.14201 PMC4988894

[30] Yang Y, Han X, Chen Y, Gao L, Yin X, Li H, Qiu J, Wang Y, Zhou Y, Xia Y. Association between modifiable lifestyle and the prevalence of atrial fibrillation in a Chinese population: based on the cardiovascular health score. Clin Cardiol 2017;40:1061–1067. 28833291 10.1002/clc.22771 PMC6490344

[31] Rienstra M, Geelhoed B, Yin X, Siland JE, Vermond RA, Mulder BA, Van Der Harst P, Hillege HL, Benjamin EJ, Van Gelder IC. Cluster individuals based on phenotype and determine the risk for atrial fibrillation in the PREVEND and Framingham heart study populations. PLoS One 2016;11:e0165828. 27832125 10.1371/journal.pone.0165828 PMC5104331

[32] Lu Y, Sun Y, Cai L, Yu B, Wang Y, Tan X, Wan H, Xu D, Zhang J, Qi L, Sanders P, Wang N. Non-traditional risk factors for atrial fibrillation: epidemiology, mechanisms, and strategies. Eur Heart J 2025;46:784–804. 39716283 10.1093/eurheartj/ehae887

[33] Kalarus Z, Mairesse GH, Sokal A, Boriani G, Sredniawa B, Casado-Arroyo R, Wachter R, Frommeyer G, Traykov V, Dagres N, Lip GYH, Boersma L, Peichl P, Dobrev D, Bulava A, Blomström-Lundqvist C, de Groot NMS, Schnabel R, Heinzel F, Van Gelder IC, Carbucicchio C, Shah D, Eckardt L. Searching for atrial fibrillation: looking harder, looking longer, and in increasingly sophisticated ways. An EHRA position paper. Europace 2023;25:185–198. 36256580 10.1093/europace/euac144 PMC10112840

