# Supplementary material for: Multimorbidity and risk of atrial fibrillation in the Lifelines cohort
Source: Eur Heart J Open. 2025 Dec 13;5(6):oeaf164. doi: 10.1093/ehjopen/oeaf164 (PMC12728500; doi:10.1093/ehjopen/oeaf164)

**Appendix I. Definitions of included risk factors and comorbidities**

**CARDIOVASCULAR COMORBIDITIES**

Hypertension

Systolic blood pressure ≥140 mmHg or diastolic blood pressure ≥90 mmHg

Heart failure

Self-reported heart failure

Diabetes mellitus

Self-reported diabetes mellitus

Hypercholesterolemia

Total cholesterol ≥5.0 mmol/L AND self-reported MI, OR total cholesterol ≥6.5 mmol/L, OR cholesterol-lowering drug use

Obesity

BMI >30 kg/m^2^

Coronary heart disease

Self-reported angioplasty OR self-reported history of myocardial infarction

Previous myocardial infarction

Self-reported history of myocardial infarction

Previous stroke

Self-reported history of stroke

Smoking status

Participants were defined as current smokers if they reported any smoking in the previous month. Participants were defined as ex-smokers if they reported that they had stopped smoking, or reported no smoking in the previous month, but reported that they had smoked for longer than a year before they had stopped smoking.

Alcohol use

Participants were asked to report how many glasses of alcoholic drinks they drink per day on average, on days that they take alcoholic drinks. We defined three groups: 1 drink or less per day, 2 drinks per day, and 3 drinks or more per day.

Physical activity

Participants were asked to report how many days per week on average they are involved in strenuous activities for at least 30 minutes, separate for summer and winter. The average of these two answers was taken as the number of days that the participant was physically active. We defined three groups: 0-1 days per week, 2-4 days per week, and 5-7 days per week.

**NON-CARDIOVASCULAR COMORBIDITIES**

Chronic obstructive pulmonary disease (COPD)

Self-reported COPD and age >40 years

Asthma

Self-reported asthma

Kidney disease

eGFR<60 ml/min/1.73m^2^

Cancer

Self-reported cancer of any type

Psoriasis

Self-reported psoriasis

Inflammatory bowel disease

Self-reported ulcerative colitis or Crohn’s disease

Rheumatoid arthritis

Self-reported rheumatoid arthritis

Osteoarthritis

Self-reported osteoarthritis

Visual impairment

Self-reported visual impairment

Migraine

Self-reported history of migraine

Dementia

Self-reported dementia or Alzheimer’s Disease

Schizophrenia

Self-reported schizophrenia

Anxiety

Any last year anxiety disorder based on the Mini International Neuropsychiatric Interview (MINI), including agoraphobia, generalised anxiety disorder, panic disorder, social phobia

Depression

Any last year depressive disorder based on the Mini International Neuropsychiatric Interview (MINI), including current depression, dysthymia, major depression

**Appendix II. Association between comorbidities and incident AF**

|  | Unadjusted | | Adjusted* | |
| --- | --- | --- | --- | --- |
| Variable | **OR (95% CI)** | **P-value** | **OR (95% CI)** | **P-value** |
| Hypertension | 2.24 (1.65-3.04) | <0.001 | 0.81 (0.59-1.11) | 0.194 |
| Heart failure | 6.79 (3.33-13.87) | <0.001 | 2.18 (1.05-4.51) | 0.037 |
| Diabetes mellitus | 4.95 (3.07-7.97) | <0.001 | 1.65 (1.01-2.69) | 0.044 |
| Hypercholesterolemia | 2.86 (2.12-3.86) | <0.001 | 1.07 (0.79-1.45) | 0.678 |
| Obesity | 2.58 (1.89-3.53) | <0.001 | 2.40 (1.75-3.30) | <0.001 |
| Coronary heart disease | 8.09 (5.21-12.56) | <0.001 | 1.41 (0.89-2.24) | 0.146 |
| Previous myocardial infarction | 6.80 (3.77-12.26) | <0.001 | 1.18 (0.64-2.17) | 0.594 |
| Previous stroke | 7.55 (3.84-14.83) | <0.001 | 2.84 (1.42-5.69) | 0.003 |
| Smoking status |  |  |  |  |
| Never | 1.00 (reference) |  | 1.00 (reference) |  |
| Ex | 2.79 (2.01-3.85) | <0.001 | 1.28 (0.92-1.79) | 0.144 |
| Current | 0.99 (0.57-1.71) | 0.977 | 1.13 (0.65-1.96) | 0.673 |
| Alcohol use |  |  |  |  |
| ≤1 glass/day | 1.00 (reference) |  | 1.00 (reference) |  |
| 2 glasses/day | 0.95 (0.67-1.35) | 0.776 | 0.90 (0.63-1.29) | 0.576 |
| ≥3 glasses/day | 1.13 (0.79-1.60) | 0.506 | 1.51 (1.03-2.20) | 0.036 |
| Physical activity |  |  |  |  |
| 0-1 days/week | 1.00 (reference) |  | 1.00 (reference) |  |
| 2-4 days/week | 0.84 (0.51-1.38) | 0.482 | 0.71 (0.43-1.17) | 0.175 |
| 5-7 days/week | 1.33 (0.84-2.11) | 0.218 | 0.69 (0.43-1.10) | 0.114 |
| COPD | 2.65 (1.63-4.31) | <0.001 | 1.40 (0.86-2.30) | 0.180 |
| Asthma | 0.86 (0.49-1.52) | 0.607 | 1.21 (0.68-2.13) | 0.516 |
| Kidney disease | 2.86 (1.59-5.14) | <0.001 | 0.79 (0.43-1.45) | 0.448 |
| Cancer | 2.05 (1.26-3.34) | 0.004 | 0.79 (0.48-1.30) | 0.349 |
| Psoriasis | 0.73 (0.27-1.96) | 0.526 | 0.63 (0.23-1.70) | 0.361 |
| Inflammatory bowel disease | 0.59 (0.08-4.22) | 0.599 | 0.70 (0.10-5.03) | 0.722 |
| Rheumatoid arthritis | 1.30 (0.54-3.17) | 0.560 | 0.71 (0.29-1.75) | 0.459 |
| Osteoarthritis | 2.53 (1.75-3.65) | <0.001 | 1.11 (0.76-1.62) | 0.596 |
| Visual impairment | 0.90 (0.61-1.33) | 0.606 | 0.81 (0.55-1.19) | 0.286 |
| Migraine | 0.75 (0.50-1.13) | 0.167 | 1.18 (0.78-1.80) | 0.437 |
| Dementia | 0.00 | 0.999 | 0.00 | 0.999 |
| Schizophrenia | 0.00 | 0.998 | 0.00 | 0.998 |
| Anxiety | 0.83 (0.45-1.53) | 0.551 | 1.19 (0.65-2.21) | 0.574 |
| Depression | 0.20 (0.03-1.42) | 0.107 | 0.35 (0.05-1.52) | 0.299 |

*Adjusted for age and sex. COPD: chronic obstructive pulmonary disease.

**Appendix III. Participant characteristics – overall and stratified by age tertiles**

| Characteristic | Total population  (n=76648) | Age tertile 1  18-41 years  (n=25934) | Age tertile 2  42-50 years  (n=25040) | Age tertile 3  51-90 years  (n=25674) |
| --- | --- | --- | --- | --- |
| Age (years) | 46.4 ± 12.6 | 32.8 ± 6.4 | 46.2 ± 2.6 | 60.2 ± 6.6 |
| Women | 45460 (59.3%) | 15939 (61.5%) | 15109 (60.3%) | 14412 (56.1%) |
| Number of risk factors and comorbidities | 2 (1–3) | 2 (1–3) | 2 (1–3) | 3 (2–4) |
| Cardiovascular | 2 (1–2) | 2 (1–2) | 2 (1–2) | 2 (1–3) |
| Non-cardiovascular | 1 (0–1) | 1 (0–1) | 1 (0–1) | 1 (0–2) |
| ≥2 Comorbidities | 56034 (73.1%) | 15828 (61.0%) | 18041 (72.0%) | 22165 (86.3% |
| ≥2 Cardiovascular comorbidities | 42575 (55.5%) | 11089 (42.8%) | 13335 (53.3%) | 18151 (70.7%) |
| ≥2 Non-cardiovascular comorbidities | 14612 (19.1%) | 2910 (11.2%) | 4789 (19.1) | 6913 (26.9%) |
| Hypertension | 13843 (18.1%) | 2129 (8.2%) | 4165 (16.6%) | 7549 (29.4%) |
| Heart failure | 505 (0.7%) | 55 (0.2%) | 97 (0.4%) | 353 (1.4%) |
| Diabetes mellitus | 1718 (2.2%) | 144 (0.6%) | 375 (1.5%) | 1199 (4.7%) |
| Hypercholesterolemia | 12457 (16.3%) | 1085 (4.2%) | 3094 (12.4%) | 8278 (32.2%) |
| Obesity | 10848 (14.2%) | 2904 (11.2%) | 3827 (15.3%) | 4117 (16.0%) |
| Coronary heart disease | 1318 (1.7%) | 41 (0.2%) | 178 (0.7%) | 1099 (4.3%) |
| Previous myocardial infarction | 771 (1.0%) | 22 (0.1%) | 107 (0.4%) | 642 (2.5%) |
| Previous stroke | 515 (0.7%) | 49 (0.2%) | 130 (0.5%) | 336 (1.3%) |
| Smoking status |  |  |  |  |
| Never | 36077 (47.1%) | 14898 (57.4%) | 12296 (49.1%) | 8883 (34.6%) |
| Ex | 28905 (37.7%) | 6177 (23.8%) | 8769 (35.0%) | 13959 (54.4%) |
| Current | 11666 (15.2%) | 4859 (18.7%) | 3975 (15.9%) | 2832 (11.0%) |

| Characteristic | Total population  (n=76648) | Age tertile 1  18-41 years  (n=25934) | Age tertile 2  42-50 years  (n=25040) | Age tertile 3  51-90 years  (n=25674) |
| --- | --- | --- | --- | --- |
| Alcohol use |  |  |  |  |
| ≤1 glass/day | 26583 (34.7%) | 7967 (30.7%) | 9039 (36.1%) | 9577 (37.3%) |
| 2 glasses/day | 27950 (36.5%) | 8137 (31.4%) | 9327 (37.2%) | 10486 (40.8%) |
| ≥3 glasses/day | 22115 (28.9%) | 9830 (37.9%) | 6674 (26.7%) | 5611 (21.9%) |
| Physical activity |  |  |  |  |
| 0-1 days/week | 9908 (12.9%) | 3810 (14.7%) | 3499 (14.0%) | 2599 (10.1%) |
| 2-4 days/week | 28525 (37.2%) | 10839 (41.8%) | 9478 (37.9%) | 8208 (32.0%) |
| 5-7 days/week | 38215 (49.9%) | 11285 (43.5%) | 12063 (48.2%) | 14867 (57.9%) |
| COPD | 2959 (3.9%) | 97 (0.4%) | 1164 (4.6%) | 1698 (6.6%) |
| Asthma | 6076 (7.9%) | 2586 (10.0%) | 1935 (7.7%) | 1555 (6.1%) |
| Kidney disease | 1793 (2.3%) | 115 (0.4%) | 379 (1.5%) | 1299 (5.1%) |
| Cancer | 3771 (4.9%) | 379 (1.5%) | 989 (3.9%) | 2403 (9.4%) |
| Psoriasis | 2229 (2.9%) | 606 (2.3%) | 739 (3.0%) | 884 (3.4%) |
| Inflammatory bowel disease | 688 (0.9%) | 192 (0.7%) | 242 (1.0%) | 254 (1.0%) |
| Rheumatoid arthritis | 1575 (2.1%) | 206 (0.8%) | 443 (1.8%) | 926 (3.6%) |
| Osteoarthritis | 6385 (8.3%) | 350 (1.3%) | 1328 (5.3%) | 4707 (18.3%) |
| Visual impairment | 13744 (17.9%) | 2873 (11.1%) | 5123 (20.5%) | 5748 (22.4%) |
| Migraine | 14003 (18.3%) | 4560 (17.6%) | 4979 (19.9%) | 4464 (17.4%) |
| Dementia | <10 (0.0%) | <10 (0.0%) | <10 (0.0%) | <10 (0.0%) |
| Schizophrenia | 55 (0.1%) | 21 (0.1%) | 27 (0.1%) | <10 (0.0%) |
| Anxiety | 5334 (7.0%) | 1850 (7.1%) | 1844 (7.4%) | 1640 (6.4%) |
| Depression | 2006 (2.6%) | 709 (2.7%) | 744 (3.0%) | 553 (2.2%) |

**Appendix IV. Logistic regression stratified by age tertiles**

|  | Age tertile 1  18-41 years | | | | Age tertile 2  42-50 years | | | | Age tertile 3  51-90 years | | | |
| --- | --- | --- | --- | --- | --- | --- | --- | --- | --- | --- | --- | --- |
|  | **Unadjusted**  **OR (95% CI)** | **P-value** | **Adjusted***  **OR (95% CI)** | **P-value** | **Unadjusted**  **OR (95% CI)** | **P-value** | **Adjusted***  **OR (95% CI)** | **P-value** | **Unadjusted**  **OR (95% CI)** | **P-value** | **Adjusted***  **OR (95% CI)** | **P-value** |
| ≥2 Comorbidities | 0.64  (0.09-4.53) | 0.654 | 0.63  (0.09-4.54) | 0.650 | 0.71  (0.26-1.92) | 0.502 | 0.58  (0.21-1.57) | 0.284 | 1.74  (1.00-3.00) | 0.049 | 1.34  (0.77-2.33) | 0.298 |
| ≥2 Cardiovascular   comorbidities | 0.45  (0.05-4.29) | 0.485 | 0.45  (0.05-4.41) | 0.490 | 1.25  (0.48-3.30) | 0.646 | 0.91  (0.34-2.42) | 0.853 | 1.98  (1.33-2.96) | <0.001 | 1.46  (0.97-2.20) | 0.068 |
| ≥2 Non-  cardiovascular   comorbidities | 0.00 | 0.987 | 0.00 | 0.986 | 1.30  (0.42-3.99) | 0.645 | 1.56  (0.50-4.82) | 0.442 | 0.88  (0.62-1.26) | 0.488 | 0.90  (0.63-1.30) | 0.581 |
| Number of comorbidities | 1.05  (0.47-2.34) | 0.903 | 1.04  (0.48-2.24) | 0.931 | 1.34  (1.02-1.75) | 0.035 | 1.28  (0.96-1.70) | 0.088 | 1.20  (1.10-1.30) | <0.001 | 1.09  (1.00-1.18) | 0.058 |
| Number of   cardiovascular   comorbidities | 1.54  (0.57-4.16) | 0.393 | 1.58  (0.59-4.25) | 0.363 | 1.81  (1.27-2.58) | 0.001 | 1.56  (1.07-2.27) | 0.020 | 1.38  (1.24-1.53) | <0.001 | 1.15  (1.03-1.28) | 0.010 |
| Number of   non-cardiovascular   comorbidities | 0.46  (0.07-3.20) | 0.435 | 0.42  (0.06-2.95) | 0.384 | 0.90  (0.53-1.54) | 0.695 | 0.98  (0.57-1.68) | 0.951 | 0.96  (0.83-1.12) | 0.605 | 0.98  (0.85-1.14) | 0.825 |
| Hypertension | 11.19  (1.58-79.49) | 0.016 | 13.33  (1.72-103.16) | 0.013 | 2.74  (1.01-7.40) | 0.047 | 1.88  (0.69-5.14) | 0.219 | 1.15  (0.83-1.59) | 0.404 | 0.72  (0.51-1.00) | 0.050 |
| Heart failure | 0.00 | 0.998 | 0.00 | 0.998 | 34.99  (7.89-155.10) | <0.001 | 30.02  (6.69-134.77) | <0.001 | 2.70  (1.19-6.15) | 0.018 | 1.64  (0.71-3.76) | 0.247 |
| Diabetes mellitus | 0.00 | 0.997 | 0.00 | 0.997 | 4.12  (0.55-31.14) | 0.170 | 3.77  (0.50-28.60) | 0.200 | 2.49  (1.52-4.07) | <0.001 | 1.60  (0.97-2.64) | 0.065 |
| Hypercholesterolemia | 0.00 | 0.992 | 0.00 | 0.992 | 2.96  (1.04-8.40) | 0.042 | 2.08  (0.73-5.94) | 0.174 | 1.24  (0.91-1.70) | 0.176 | 1.01  (0.73-1.39) | 0.953 |
| Obesity | 7.94  (1.12-56.35) | 0.038 | 6.49  (0.90-46.73) | 0.063 | 3.89  (1.45-10.21) | 0.006 | 3.97  (1.51-10.45) | 0.005 | 2.06  (1.47-2.90) | <0.001 | 2.19  (1.55-3.09) | <0.001 |

|  | Age tertile 1  18-41 years | | | | Age tertile 2  42-50 years | | | | Age tertile 3  51-90 years | | | |
| --- | --- | --- | --- | --- | --- | --- | --- | --- | --- | --- | --- | --- |
|  | **Unadjusted**  **OR (95% CI)** | **P-value** | **Adjusted***  **OR (95% CI)** | **P-value** | **Unadjusted**  **OR (95% CI)** | **P-value** | **Adjusted***  **OR (95% CI)** | **P-value** | **Unadjusted**  **OR (95% CI)** | **P-value** | **Adjusted***  **OR (95% CI)** | **P-value** |
| Coronary heart disease | 0.00 | 0.998 | 0.00 | 0.998 | 0.00 | 0.996 | 0.00 | 0.996 | 3.63  (2.33-5.66) | <0.001 | 1.46  (0.92-2.33) | 0.111 |
| Previous myocardial infarction | 0.00 | 0.999 | 0.00 | 0.999 | 0.00 | 0.997 | 0.00 | 0.997 | 3.06  (1.69-5.53) | <0.001 | 1.22  (0.66-2.24) | 0.528 |
| Previous stroke | 0.00 | 0.998 | 0.00 | 0.998 | 0.00 | 0.997 | 0.00 | 0.997 | 4.39  (2.22-8.66) | <0.001 | 2.96  (1.47-5.93) | 0.002 |
| Smoking status |  |  |  |  |  |  |  |  |  |  |  |  |
| Never | 1.00  (reference) |  | 1.00  (reference) |  | 1.00 (reference) |  | 1.00  (reference) |  | 1.00 (reference) |  | 1.00  (reference) |  |
| Ex | 0.00 | 0.981 | 0.00 | 0.980 | 0.42  (0.12-1.53) | 0.188 | 0.42  (0.11-1.52) | 0.185 | 1.79  (1.25-2.56) | 0.001 | 1.44  (1.00-2.08) | 0.051 |
| Current | 3.07  (0.43-21.78) | 0.262 | 3.37  (0.47-24.08) | 0.226 | 1.24  (0.39-3.95) | 0.719 | 1.13  (0.35-3.61) | 0.837 | 0.84  (0.43-1.64) | 0.611 | 0.98  (0.50-1.93) | 0.961 |
| Alcohol use |  |  |  |  |  |  |  |  |  |  |  |  |
| ≤1 glass/day | 1.00  (reference) |  | 1.00  (reference) |  | 1.00 (reference) |  | 1.00  (reference) |  | 1.00 (reference) |  | 1.00  (reference) |  |
| 2 glasses/day | 0.00 | 0.976 | 0.00 | 0.976 | 0.969  (0.20-4.80) | 0.969 | 0.73  (1.15-3.65) | 0.699 | 0.96  (0.67-1.38) | 0.826 | 0.96  (0.66-1.38) | 0.809 |
| ≥3 glasses/day | 0.27  (0.03-2.60) | 0.257 | 0.41  (0.03-4.96) | 0.486 | 4.97  (1.39-17.83) | 0.014 | 2.70  (0.72-10.22) | 0.143 | 1.42  (0.97-2.08) | 0.076 | 1.44  (0.95-2.17) | 0.086 |

|  | Age tertile 1  18-41 years | | | | Age tertile 2  42-50 years | | | | Age tertile 3  51-90 years | | | |
| --- | --- | --- | --- | --- | --- | --- | --- | --- | --- | --- | --- | --- |
|  | **Unadjusted**  **OR (95% CI)** | **P-value** | **Adjusted***  **OR (95% CI)** | **P-value** | **Unadjusted**  **OR (95% CI)** | **P-value** | **Adjusted***  **OR (95% CI)** | **P-value** | **Unadjusted**  **OR (95% CI)** | **P-value** | **Adjusted***  **OR (95% CI)** | **P-value** |
| Physical activity |  |  |  |  |  |  |  |  |  |  |  |  |
| 0-1 days/week | 1.00  (reference) |  | 1.00  (reference) |  | 1.00 (reference) |  | 1.00  (reference) |  | 1.00 (reference) |  | 1.00  (reference) |  |
| 2-4 days/week | 0.35  (0.02-5.62) | 0.460 | 0.37  (0.02-5.95) | 0.484 | 0.31  (0.09-1.01) | 0.051 | 0.29  (0.09-0.94) | 0.039 | 0.99  (0.55-1.78) | 0.979 | 0.89  (0.50-1.60) | 0.700 |
| 5-7 days/week | 0.68  (0.06-7.45) | 0.748 | 0.69  (0.06-7.65) | 0.765 | 0.29  (0.09-0.90) | 0.032 | 0.28  (0.09-0.88) | 0.029 | 1.23  (0.71-2.11) | 0.463 | 0.84  (0.49-1.46) | 0.536 |
| COPD | 0.00 | 0.998 | 0.00 | 0.997 | 2.74  (0.63-11.99) | 0.181 | 2.67  (0.61-11.70) | 0.193 | 1.50  (0.89-2.52) | 0.124 | 1.32  (0.78-2.23) | 0.298 |
| Asthma | 0.00 | 0.987 | 0.00 | 0.987 | 1.59  (0.36-6.97) | 0.537 | 1.68  (0.38-7.37) | 0.491 | 1.09  (0.59-2.02) | 0.773 | 1.20  (0.65-2.23) | 0.562 |
| Kidney disease | 0.00 | 0.997 | 0.00 | 0.997 | 0.00 | 0.995 | 0.00 | 0.995 | 1.46  (0.81-2.63) | 0.211 | 0.82  (0.45-1.50) | 0.517 |
| Cancer | 0.00 | 0.995 | 0.00 | 0.995 | 1.52  (0.20-11.48) | 0.685 | 1.78  (0.24-13.57) | 0.576 | 1.10  (0.66-1.82) | 0.715 | 0.77  (0.46-1.29) | 0.318 |
| Psoriasis | 0.00 | 0.994 | 0.00 | 0.994 | 0.00 | 0.992 | 0.00 | 0.992 | 0.69  (0.25-1.86) | 0.459 | 0.70  (0.26-1.91) | 0.487 |
| Inflammatory bowel disease | 0.00 | 0.997 | 0.00 | 0.996 | 0.00 | 0.996 | 0.00 | 0.996 | 0.60  (0.08-4.31) | 0.613 | 0.79  (0.11-5.70) | 0.816 |
| Rheumatoid arthritis | 0.00 | 0.996 | 0.00 | 0.996 | 3.48  (0.46-26.27) | 0.227 | 3.55  (0.47-26.64) | 0.220 | 0.65  (0.24-1.77) | 0.403 | 0.59  (0.22-1.61) | 0.305 |
| Osteoarthritis | 0.00 | 0.995 | 0.00 | 0.995 | 0.00 | 0.990 | 0.00 | 0.989 | 1.18  (0.81-1.72) | 0.380 | 1.15  (0.78-1.68) | 0.483 |

|  | Age tertile 1  18-41 years | | | | Age tertile 2  42-50 years | | | | Age tertile 3  51-90 years | | | |
| --- | --- | --- | --- | --- | --- | --- | --- | --- | --- | --- | --- | --- |
|  | **Unadjusted**  **OR (95% CI)** | **P-value** | **Adjusted***  **OR (95% CI)** | **P-value** | **Unadjusted**  **OR (95% CI)** | **P-value** | **Adjusted***  **OR (95% CI)** | **P-value** | **Unadjusted**  **OR (95% CI)** | **P-value** | **Adjusted***  **OR (95% CI)** | **P-value** |
| Visual impairment | 0.00 | 0.987 | 0.00 | 0.986 | 0.83  (0.24-2.90) | 0.774 | 0.76  (0.22-2.67) | 0.671 | 0.70  (0.46-1.05) | 0.082 | 0.82  (0.54-1.24) | 0.343 |
| Migraine | 1.56  (0.16-15.03) | 0.699 | 1.27  (0.13-12.64) | 0.839 | 0.25  (0.03-1.90) | 0.181 | 0.39  (0.05-2.95) | 0.359 | 0.84  (0.55-1.28) | 0.409 | 1.28  (0.83-1.98) | 0.269 |
| Dementia | 0.00 | 1.000 | 0.00 | 1.000 | 0.00 | 1.000 | 0.00 | 1.000 | 0.00 | 0.999 | 0.00 | 0.999 |
| Schizophrenia | 0.00 | 0.999 | 0.00 | 0.999 | 0.00 | 0.999 | 0.00 | 0.998 | 0.00 | 0.999 | 0.00 | 0.999 |
| Anxiety | 0.00 | 0.989 | 0.00 | 0.989 | 1.68  (0.38-7.34) | 0.92 | 2.14  (0.49-9.42) | 0.314 | 0.83  (0.43-1.64) | 0.597 | 1.13  (0.57-2.23) | 0.723 |
| Depression | 0.00 | 0.993 | 0.00 | 0.993 | 0.00 | 0.992 | 0.00 | 0.992 | 0.27  (0.04-1.95) | 0.195 | 0.44  (0.06-3.13) | 0.408 |

*****Adjusted for age and sex

**Appendix V.**

Figure demonstrating the cardiovascular and non-cardiovascular comorbidities captured in the study. Those marked in red, denote the additional comorbidities which were not available in this analysis.

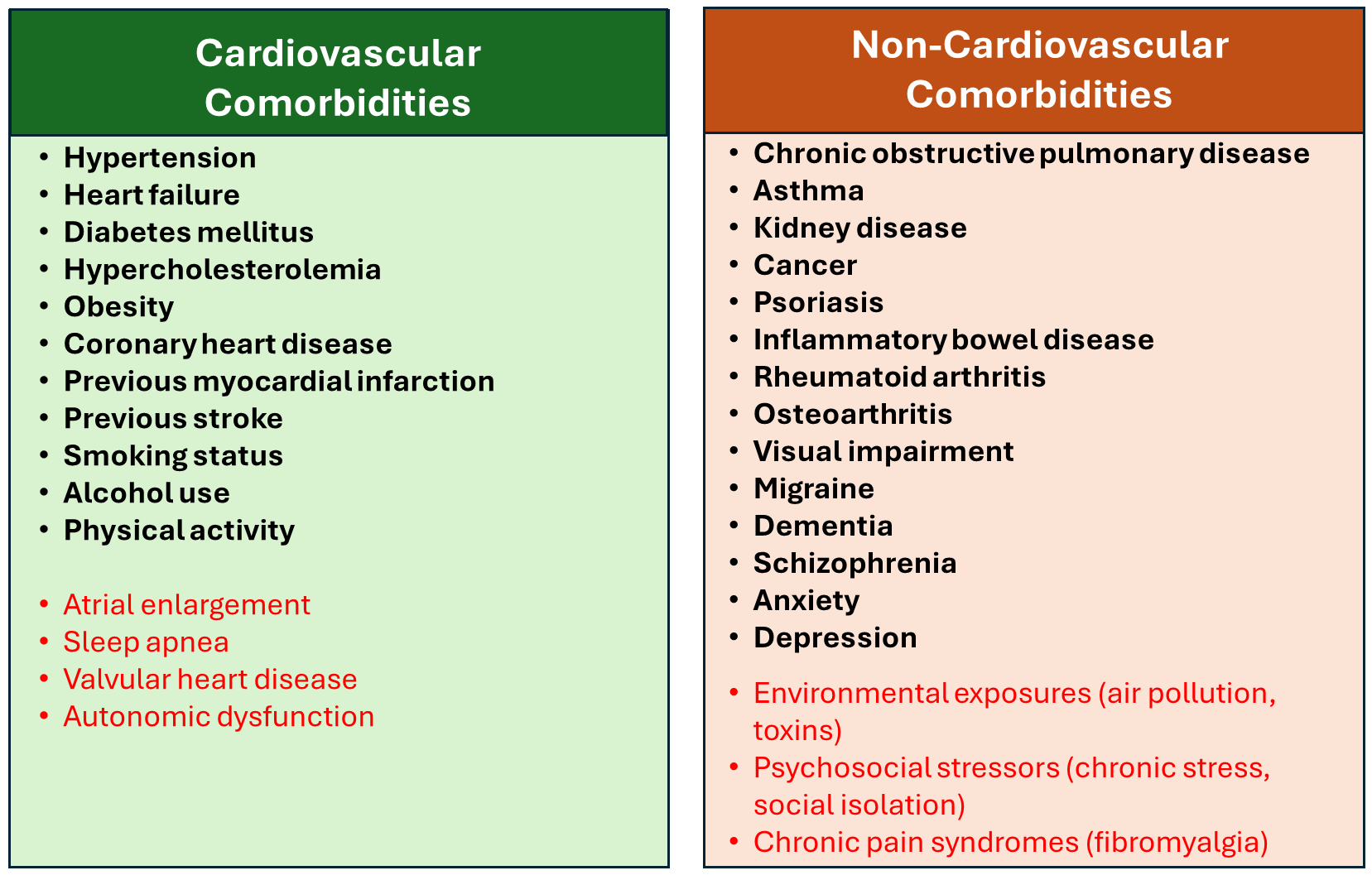

Supplement: oeaf164_Supplementary_Data [file oeaf164_supplementary_data.docx]
